# Perception of depressive symptoms by the Sardinian public: results of a population study

**DOI:** 10.1186/1471-244X-13-57

**Published:** 2013-02-16

**Authors:** Mauro Giovanni Carta, Matthias C Angermeyer, Herbert Matschinger, Anita Holzinger, Francesca Floris, Maria Francesca Moro

**Affiliations:** 1 Department of Public Health and Clinical and Molecular Medicine, University of Cagliari, Cagliari, Italy; 2 Center for Public Mental Health, Gösing am Wagram, Austria; 3 Institute of Social Medicine, Occupational Health and Public Health, University of Leipzig, Leipzig, Germany; 4 Institute of Medical Sociology, Social Medicine and Health Economics, University of Hamburg, Hamburg, Germany; 5 Department of Psychiatry and Psychotherapy, Medical University of Vienna, Vienna, Austria

**Keywords:** Diagnosis, Help-seeking, Major depressive disorder, Population study

## Abstract

**Background:**

With the exception of bereavement, the diagnosis of major depressive disorder in the DSM-IV does not take into account the context in which symptoms occur. Recent criticism has maintained that common sense suggests making a distinction between depression as mental disorder and sorrow as ‘normal’ reaction to social stress. Results of a study from Vienna support this view. This study sets out to examine whether these results can be replicated in a different cultural setting.

**Methods:**

In 2012, a population-based survey was conducted by phone in Sardinia (n = 1,200). A fully structured interview was carried out which began with the presentation of a vignette depicting a diagnostically unlabeled case of depression, with or without provision of information about preceding stressful life events.

**Results:**

In general, as compared to the people from Vienna, the Sardinian public was much less prone to define depressive symptoms as expression of mental illness and more reluctant to recommend professional help. However, similar to Vienna, respondents presented with vignettes containing information on loss events were less likely to define depressive symptoms as indication of a psychiatric illness. They were also less willing to recommend professional help and relied more on self-help and support by family members and close friends.

**Conclusions:**

We were able to replicate the result of the previous study that the public tends to perceive depressive symptoms differently depending on the context in which they occur. This lets us conclude that the divide between the public’s view of what depression is and the view of DSM-IV is not limited to a particular culture but seems to represent a more general phenomenon. In consequence, one might rethink the diagnostic criteria for major depressive disorder in order to reconcile both views.

## Background

The diagnosis of major depression in the DSM-IV requires the presence of at least five symptoms from a list of nine, including sadness or lack of interest or pleasure in life, at least two weeks’ duration and clinically significant impairment or distress. The context in which the symptoms occur is not taken into account; only the clinical picture counts. The sole exception to the rule is represented by bereavement. In this case, people who otherwise meet the symptomatic descriptions for major depressive disorder should nevertheless not receive this diagnosis, unless more than two months elapsed since the death or certain other severe symptoms or marked impairment are present [1]. However, for the forthcoming DSM-5 it has been proposed to eliminate this exclusion on the grounds that empirical evidence would not justify a special diagnostic status [2]. Based on a re-analysis of recent studies that are claimed to support the DSM-5 proposal this has been contested by Wakefield & First (2012) who argue that there is no scientific evidence to support diagnosing as major depression two weeks of grief-related depression feelings [3].

The “de-contextualized” approach of DSM has recently been criticized by Horwitz & Wakefield (2007) [4]. In their book entitled “The Loss of Sadness” they stress the importance of distinguishing between abnormal reactions because of internal dysfunction and normal sadness brought on by external circumstances. Under the current DSM classification system, this distinction is impossible to make. The authors maintain that expected emotional distress caused by upsetting events such as the end of a romantic relationship or the loss of a job could lead to a mistaken diagnosis of depressive disorder. According to them, it is this “conflation of normality and pathology” that lies at the root of the presumed epidemic of major depression that has recently been reported. On several occasions the authors refer to common sense that would suggest discriminating between sorrow and depression.

Findings from a population survey that has been carried out in Vienna in 2009 support in fact the view that the public tends to perceive depressive symptoms differently depending on the context in which they occur. Respondents presented with vignettes containing information on loss events were less likely to define depressive symptoms as indication of mental illness. They were also not as willing to recommend professional help. As the authors correctly point out, before generalizing these findings, replication with other populations is necessary [5].

We, therefore, set out to conduct a similar study in another cultural setting. We have chosen Sardinia (Italy) which differs from Vienna in many respects: Vienna, capital city of Austria, is a large, metropolitan city with almost two million inhabitants, the cradle of psychoanalysis, with a high density of mental health professionals of all kinds. By contrast, half of the Sardinian population lives in small communities with less than 5,000 inhabitants many of them located in remote areas with insufficient provision of secondary health services. Unlike the Viennese, quite a few Sardinians, particularly those living in rural areas, still tend to adhere to old traditions. As an example may serve practices aimed at protecting people from supernatural powers, for instance the folk custom to leave a cup of milk on the table when there is a newborn in the house in order to keep away the surbile (kind of a witch that can transform itself into a cat or bat which may drink the blood of the child), or that children may not be allowed to leave the house during strong sun-shine in order to prevent them being kidnapped by the mammai e su sole (an ugly old woman) [6-8].

We were interested in knowing whether what has been found in Vienna can be replicated in Sardinia. Specifically, we wanted to test the following hypotheses: Respondents who have been presented with a vignette containing information on negative life events (1) are less likely to define depressive symptoms as an indication of mental illness, and (2) will less likely endorse professional help than respondents who have been presented with a vignette containing no such information.

## Methods

### Sample

From November 2011 to August 2012, a population-based survey was conducted by phone in Sardinia, involving persons aged 16 years and older, living in private households with conventional telephone connection. For sampling, the Sardinian population was broken down into 16 geographically defined strata representing the capital cities of the eight provinces (Cagliari, Carbonia-Iglesias, Nuoro, Ogliastra, Olbia-Tempio, Oristano, Sassari, Villacidro-Sanluri) plus villages with less than 5,000 inhabitants within each province. These strata were then subdivided into ten cells according to sex and five age groups (16–29, 30–44, 45–59, 60–74, 75+). A random sample was drawn from all registered private telephone numbers and additionally generated numbers, allowing for extra-directory households as well. As soon as a cell was saturated, persons who did not meet age and sex criteria were excluded from the data base. 1,200 persons, i.e. 51% of those who were eligible for interview (N= 2343), agreed to participate in the study. The interviews were carried out by medical doctors, psychologists and post-graduate students in mental health and primary care.

### Ethics

Informed consent was considered to be given when the person agreed to the interview. Data were not nominal at the source, and each subject was identified with a code number. The survey was approved by the ethics committee of the Azienda Mista Ospedaliero-Universitaria, Cagliari. The research was conducted in compliance with the Helsinki Declaration.

### Interview

Computer assisted telephone interviewing (CATI) was used. The fully-structured interview was identical to the one developed for the study in Vienna [5]. The interview was translated into Italian following the guidelines of WHO [9]. It began with the presentation of a vignette describing a diagnostically unlabeled case of depression fulfilling the criteria of DSM-IV for a moderate major depression. Independently drawn subsamples of ca. 300 persons were presented with one of four different versions of the case history. The first version provided a pure description of depressive symptoms plus information on duration and impairment of functioning; the other informations about life events that had preceded the depressive episode: death of spouse, betrayal by the romantic partner, and loss of work (see Appendix for exact wording of the vignettes). The gender in the vignettes was varied at random. For maximum standardization of the stimulus, the case vignettes were pre-recorded with a male and a female voice, and for each interview, one of the two recordings was chosen at random to be played to the respondent.

After presentation of the vignette the respondents were asked whether they considered the person depicted in the vignette as being mentally ill (“Would you say, this person suffers from a mental illness in a medical sense?”, response categories yes/no/don’t know). This was followed by the question “What should be done in such a situation? Which of the following three options would you recommend?”. Respondents had to choose between “do nothing and wait”, “try to do something against it oneself or seeking support from family members or close friends”, or “seek professional help”. If the first option had been chosen, respondents then were asked: “If the condition hasn’t changed after a few months, what would you now recommend?”, offering the same three options. If the respondents had opted for the second possibility they had to choose between the alternatives “continue trying to do something against it oneself” and “seeking professional help”.

Before starting fieldwork, the interview was pilot-tested with 20 randomly chosen lay persons. Interviewer training as well as field work was supervised by MFM.

### Statistical analysis

To test the first hypothesis, multinomial logit models were estimated with the type of vignette as independent variable and the definition of the condition depicted in the vignette as mental illness as dependent variable, holding respondents’ gender, age, familiarity with treatment for depression (respondent, family or friends), contact at work with people with mental health problems, depressive symptoms as assessed by the mood subscale of the Patient Health Questionnaire (PHQ-9, Italian version [10]), and the gender of the person depicted in the vignette, constant. For testing the second hypothesis, the same procedure was chosen, this time using the respondents’ recommendations as to what should be done in this situation as dependent variable.

To illustrate the magnitude of differences between the subsamples exposed to the different vignette versions, discrete probability changes were calculated for all items and each response category. A discrete change coefficient is the difference in the predicted probability of a given outcome between the various subsamples. 95% confidence intervals were computed with the delta method. To make adjusted predictions comparable to unadjusted survey results, probabilities and discrete changes are multiplied by 100 and can be read as percentages of respondents choosing any answer category. An advantage of discrete probability changes is that they are independent from the criterion used as reference category. They also give an idea of the magnitude of the effect and can serve as indicator of the effect size of the change. The calculation of probability changes and the testing for differences in probabilities between subsamples were carried out using the modules prvalue and prchange [11,12] in STATA [13].

## Results

### Characteristics of the sample

The socio-demographic characteristics of our sample are reported in Table 1. For comparison, data for the general population of Sardinia are also shown. As can be seen, the sample characteristics are reasonable consistent with those of the general population. Of the respondents, 10.8% had already been in treatment themselves because of depression, 41.8% had someone in the family or among their close friends with a history of treatment for depression, and 32.2% had contact within the context of their profession with people with mental health problems. The mean PHQ score was 3.9 (SD 3.8).

**Table 1:** Socio-demographic characteristics of sample

	**Sample (N=1,200) %**	**Total population of Sardinia (N=1,675,411) %**
Gender		
Female	51.5	50.9
Male	48.5	49.0
Age (years)		
16–29	18.5	17.6
30–44	27.7	26.9
45–59	25.0	25.7
60–74	18.8	19.2
75+	10.0	10.5
Residency (provinces)		
Cagliari	32.9	32.7
Carbonia-Iglesias	7.6	8
Nuoro	10.0	9.8
Ogliastra	3.6	3.7
Olbia-Tempio	9.3	9.2
Oristano	10.0	10.4
Sassari	20.3	20.1
Villacidro-Sanluri	6.3	6.1

Hypothesis 1: Respondents presented with a vignette containing information on negative life events are less likely to define depressive symptoms as an indication of a mental illness in a medical sense.

**Figure 1:**
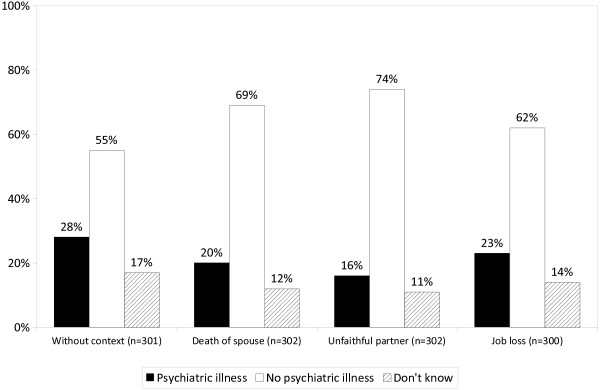
**The public’s definition of depressive symptoms as indication of a psychiatric illness in the medical sense. **Question: “Does this person suffer from a psychiatric illness in a medical sense?”.

As shown in Figure 1, across all four vignettes only a minority (16% - 28%) defined the condition described in the vignette as mental illness and the majority answered in the negative (55% - 73%). However, when the depressive symptoms where preceded by the death of spouse or occurred in the context of the betrayal by a romantic partner, the probability that the illness concept was endorsed was significantly lower than when the depressive symptoms were presented without context (probability change -9% or -13%, respectively). Correspondingly, the percentage of respondents who did not define depressive symptoms as an indication of a psychiatric illness increased by 13% or 18%, respectively. With the vignette showing depressive symptoms following job loss, the difference to the vignette without context was not significant (Table 2).

**Table 2:** The public’s definition of depressive symptoms as indication of a psychiatric illness, depending on type of vignette (multinomial logit regression)

	**Predicted percentages**				**Probability change**					
	**Without context**	**Death of spouse**	**Unfaithful partner**	**Job loss**	**Change 1-2**^**b**^	**Change 1-3**^**b**^	**Change 1-4**^**b**^	**Change 2-3**^**b**^	**Change 2-4**^**b**^	**Change 3-4**^**b**^
	**(N=292)**^**a**^	**(N=293)**^**a**^	**(N=281)**^**a**^	**(N=281)**^**a**^						
	**1**	**2**	**3**	**4**						
Psychiatric illness	28	19	15	23	**-9 (-16,-2)**	**-13 (-20,-6)**	-6 (-13,1)	-4 (-10,2)	3 (-3,10)	**7 (1,14)**
No psychiatric illness	56	69	74	63	**13 (5,21)**	**18 (11,26)**	7 (-1,16)	5 (-2,13)	-6 (-14,2)	**-11 (-19,-3)**
Don’t know	16	12	10	14	-4 (-10,1)	-5 (-11,1)	2 (-7,4)	-1 (-6,4)	3 (-3,8)	4 (-2,9)

^a ^N less than 300 due to missing values.

^b ^Due to rounding figures shown will not always equal the difference between predicted percentages for the public’s definition of depressive symptoms.

### Hypothesis 2: Respondents presented with a vignette containing information on negative life events are less likely to recommend professional help for depressive symptoms

**Figure 2:**
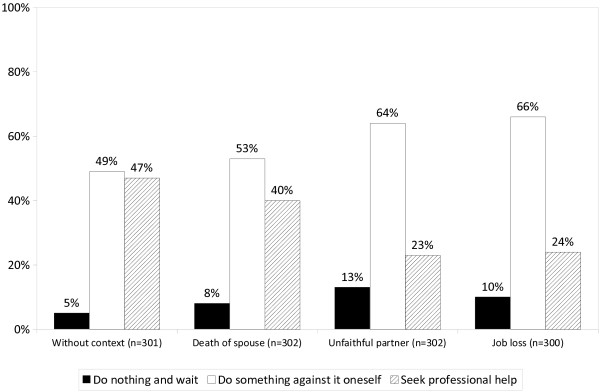
**The public’s recommendations of what should be done in case of depressive symptoms (1). **Question: “What should be done in such a situation?”.

When asked what should be done in such a situation, almost half of those presented with the vignette without context endorsed self-help or support by family members or friends and almost half recommended seeking professional help. Merely 5% recommended doing nothing and waiting. While respondents’ recommendations were only slightly and not significantly different when presented with the vignette depicting depressive symptoms following the death of the spouse, there were marked differences concerning the other two life events. In the case of betrayal by a romantic partner 64% recommended self-help or turning to someone close and only 23% seeking help from a professional. When compared with the vignette without context, the probability of seeking professional help being recommended dropped significantly by 26%, while the probability that self-help was recommended increased by 17%. Practically identical were the results for the vignette showing depressive symptoms after job loss, where the probability that seeking professional help was recommended dropped by 24% and, correspondingly, the probability that relying on self-help or support by family members or friends increased by 18% (Figure 2, Table 3).

**Table 3:** The public’s recommendation of what should be done in case of depressive symptoms (multinomial logit regression)

	**Predicted percentages**				**Probability change**					
	**Without context**	**Death of spouse**	**Unfaithful partner**	**Job loss**	**Change 1-2**^**b**^	**Change 1-3**^**b**^	**Change 1-4**^**b**^	**Change 2-3**^**b**^	**Change 2-4**^**b**^	**Change 3-4**^**b**^
	**(N=292)**^**a**^	**(N=293)**^**a**^	**(N=281)**^**a**^	**(N=281)**^**a**^						
	**1**	**2**	**3**	**4**						
Do nothing and wait	5	7	13	10	3 (-1,7)	**9 (4,13)**	**5 (1,10)**	**6 (1,11)**	2 (-2,9**)**	-3 (-9,2)
Do something against it oneself	48	53	64	67	5 (-4,13)	**17 (9,25)**	**18 (11,27)**	**12 (1,20)**	**14 (3,22)**	2 (-6,10)
Seek professional help	47	40	22	23	-8 (-16,0)	**-26 (-33,-18)**	**-24 (-32,-16)**	**-18 (-25,-10)**	**-16 (-23,-8)**	1 (-6,8)

^a ^N less than 300 due to missing values.

^b ^Due to rounding figures shown will not always equal the difference between predicted percentages for the public’s recommendations of what should be done.

**Figure 3:**
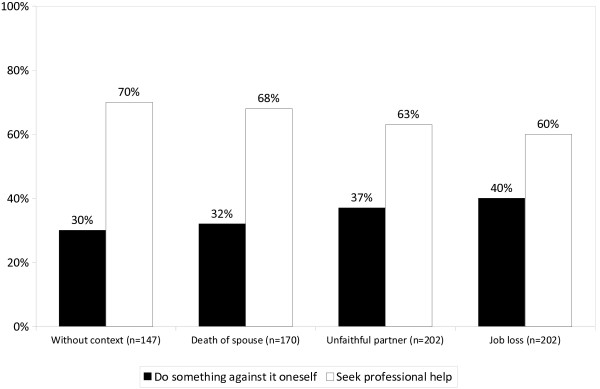
**The public’s recommendations of what should be done in case of depressive symptoms (2). **Question: “If the condition remains unchanged after several months, what would you recommend next?”.

As described in the Method section, respondents who had endorsed self-help or seeking help from family or friends were asked again what they would recommend if the condition had not changed after several months. Now, the situation changed substantially in that across all four vignettes more respondents (60% - 70%) opted for professional help and fewer for self-help or help from someone close (30% - 40%). While there was no difference between the vignette without context and the vignette with death of spouse, slightly more respondents tended to continue to endorse self-help or support by family or friends when presented with the vignettes where depressive symptoms occur in the context of betrayal by a romantic partner or job loss (probability change 10% or 12%, respectively) (Figure 3).

### Variation in the perception of depressive symptoms according to the preceding life events

As concerns the definition of depressive symptoms as an indication of psychiatric illness respondents made no distinction between death of spouse and the other two life events. However, there was a statistically significant difference between betrayal by the romantic partner and job loss, with the first less frequently being considered a psychiatric illness (probability change -11%). With regard to the three different options of dealing with depressive symptoms, the public’s choices were similar in case of the unfaithful partner and job loss, which both differed significantly from death of spouse where respondents opted less frequently for self-help or help by someone close (probability change 12% and 14%, respectively) and more frequently for professional help (probability change -18% and -16%, respectively) (Tables 2 and 3).

### Relationship between the definition of depressive symptoms and recommendation of professional help

Comparing Figure 1 and 2 one notices that more respondents endorsed professional help than judged depressive symptoms as indication of a mental illness. In fact, 34% of those presented with the vignette without preceding life event, who had not defined depressive symptoms as sign of mental illness, recommended seeking professional help. A similar percentage (30%) was found when depressive symptoms occurred after loss of partner while in case of betrayal by a romantic partner and job loss only a small minority of those who had disagreed with the mental illness label recommended professional help at the same time (17% and 13%, respectively). Irrespective of that, across all four vignettes professional help was recommended more frequently if respondents defined depressive symptoms as indication of mental illness than if they did not. The difference was particularly pronounced in case of betrayal of a romantic partner (55% vs. 17%) and job loss (56% vs. 13%), while with bereavement it was 70% vs. 30%, and in case of no preceding life event 73% vs. 34%.

## Discussion

In support of our hypotheses, depressive symptoms occurring in conjunction with the loss of someone close, because of death of the spouse or because betrayal by a romantic partner, were more frequently not seen as indication of a psychiatric illness than when they occurred without preceding negative life events. Although there was a tendency into the same direction, no statistically significant difference was observed with job loss. As predicted, in case of betrayal by a romantic partner and job loss respondents were also less ready to recommend seeking professional help while in case of death of spouse the difference to the vignette without context did not reach statistical significance. Thus, with the exception of labeling job loss, we were able to replicate all results of the Vienna study, not only as concerns the differences between vignettes with and without negative life events but also as concerns the differences within vignettes with preceding life events. What has been observed in Vienna could also be observed in a quite different cultural setting. This is an important finding as it indicates that making a distinction between depression as a disorder and sorrow as a ‘normal’ reaction to social stress is not a peculiarity of the people from Vienna but something that can be found across different cultures. Obviously, from the public’s perspective the context in which depressive symptoms occur matters. This lends further support to Horwitz & Wakefield’s [4] argument that when depressive symptoms occur in the context of adverse life events, the diagnosis of major depressive disorder should not automatically be made.

While the pattern across vignettes of defining depressive disorders was quite similar in Sardinia and Vienna there were huge differences with regard to the degree to which depressive symptoms were considered in general as an indication of psychiatric illness. Across all four case-vignettes, the proportion of respondents endorsing an illness concept was in Vienna over twice as high as in Sardinia. In Vienna, 67% of respondents presented with the vignette without context considered depressive symptoms as expression of a psychiatric illness, as compared to only 28% in Sardinia. The respective percentages for the vignette with death of spouse were 42% vs. 20%, for the vignette with betrayal of a romantic partner 39% vs. 16%, and for the vignette with job loss 52% vs. 23%. That in Sardinia the conceptualization of depression as a medical illness is much less popular than in Vienna may be explained in different ways. Some Sardinians may still adhere to a more traditional understanding of psychic suffering rather than endorse modern explanations of mental health problems in terms of biological or psychological mechanisms. For instance, s’ogu pigau or s’oglu pigau (captivated by the look of an envious person) was known to be the cause of mental health problems like loss of energy, sadness and loss of interest [14]. Although this concept nowadays may only be present in villages in remote areas of the island, there may be still a certain inclination to interpret depressive symptoms in more traditional ways, the more as the Sardinian culture is well known for its “resistance” against “exterior values and innovations” and its “neurotic” emphasis on tradition [15]. Another point which may play a part is that it was relatively late that psychiatry has found its way into Sardinia. For instance, the first professor of psychiatry was appointed at the University of Cagliari, the capital city of Sardinia, in 1956, long after the first psychiatrist had been awarded the “venia legendi” from the University of Vienna (in 1843), and Theodor Meynert had there become the first chair of psychiatry (in 1879). While in Vienna the first institution to accommodate mental patients (“Narrenturm” , “Fools’ Tower”) was built in 1784, the first psychiatric hospital in Sardinia was opened in Cagliari as late as in 1904 [16]. In the province of Nuoro, in the inner part of the island, there has never been a hospital providing inpatient care for mentally ill people from the area. This also may help understand the relatively limited impact of psychiatry on lay conceptualizations of mental disorder. Taking into account that in recent years strong efforts have been made to promulgate the notion that depression is the most common mental disorder [17], representing one of the most important contributors to the burden of disease worldwide [18], the reluctance of the Sardinian people to endorse the medical model of depression is the more remarkable.

In view of their reluctance to endorse medical concepts of mental illness it comes as no surprise that, when asked what action should be taken to overcome depressive symptoms, people from Sardinia tended less to opt for professional help than people from Vienna. This held true for all four vignettes. For instance when confronted with the vignette without context, in Vienna 61.5% of respondents recommended turning to a professional for help while in Sardinia there were only 47% who had chosen this option. By contrast, in Sardinia half of respondents preferred relying on self-help or support by family members or close friends while in Vienna only slightly over one third endorsed this coping strategy. If the condition had not changed after several months, in Vienna hardly anybody, just 4% of respondents, stuck to self-help or seeking help from family members of close friends while in Sardinia still 30% were giving it priority over professional help. Similar differences were also found with the other vignettes. Obviously, the inclination to rely on oneself or one’s own family as well as the reservation against using professional help was much more pronounced among the Sardinian people. Apart from what has been discussed above this may also be explained by the Sardinian culture which is heavily centered on individualism and self-efficacy (“balentia”) [19]. In this context, seeking help from someone else may be looked at as a sign of weakness, resulting in the stigmatization of such behavior. Another reason for Sardinians’ stronger reliance on self-help may simply be the fact that, as already mentioned in the Introduction, psychosocial services are rather scarce on the island, particularly on the countryside [20]. Thus, even if people had seen the need for professional help they might not have opted for utilizing such services taking into account their poor availability.

Our findings document that in the eyes of the public both “diagnosis” of mental illness and perception of the need for professional help are not as tightly linked as it is posited by professionals. Particularly when symptoms occur without preceding life events, labeling as mental illness is no necessary precondition for perceiving a need for professional help. Similar disparities in lay judgments of illness status and need-for-help judgments have also been reported for conduct disorders [21]. This is an important point as it helps separate issues of service provision from the necessity of a diagnosis, at least if looked at it from a lay perspective. On the other hand our findings show that the definition as mental illness is taken as an indicator of the need for professional care, particularly when life events like betrayal by a romantic partner or job loss precede the onset of depressive symptoms. Thus, whether or not people endorse the mental illness label has consequences for the choice of actions to be taken.

Some limitations of our study need mentioning. First, using vignettes as stimulus, we cannot exclude that, if confronted with a similar situation in real life, respondents might have reacted somewhat differently. Second, the impact of only three negative life events on the public’s perception of depressive symptoms has been investigated. It would be interesting to also include other life events such as the diagnosis of a serious illness in oneself or a loved one. The strength of our study is that pains were taken to adhere as closely as possible to the design of the study in Vienna by using the same sampling procedure (quota sampling), interview mode (CATI), and the same vignettes and the same interview, which allows direct comparison of results from both studies.

## Conclusion

In conclusion we can state that similarities as well as differences emerged from the comparison between Sardinia and Vienna. As compared to the people from Vienna, Sardinians were in general much less prone to interpret depressive symptoms as expression of mental illness and more reluctant to recommend professional help. There seems to be a need for raising the awareness of the Sardinian public that depressive symptoms may be an indication of psychiatric disorder in need of professional treatment. It seems also necessary to combat the stigma surrounding seeking professional help in case of mental illness.

Similar to Vienna, respondents presented with vignettes containing information on loss events were less likely to define depressive symptoms as indication of a psychiatric illness. They were also less willing to recommend professional help and relied more on self-help and support by family members and close friends. Thus, the divide between the public’s view of what depression is and the view of DSM-IV did exist in both Sardinia and Vienna. It is not limited to a particular culture but seems to represent a more general phenomenon. As Mario Maj has recently pointed out “it appears, therefore, urgent to articulate a convincing response to the question “When does depression become a mental disorder?” [22], One might rethink the diagnostic criteria for major depressive disorder in order to reconcile both views. It may be seen as a first step in this direction that the DSM-5 Mood Disorders Work-group, which had previously recommended the elimination of the bereavement exclusion criteria from major depressive episodes, is now proposing (along with removing the exclusion) the addition of a footnote to accompany the diagnostic criteria for Major Depressive Episode, that would clarify the difference between the normal and expected response to an event involving significant loss (e.g., bereavement, financial ruin, natural disaster) and the diagnosis of Major Depression [23].

## Appendix

Vignettes (female version)

Major depressive disorder as in DSM-IV

For several weeks, a friend of yours has been practically always depressed and sad. She cannot laugh about anything anymore. She is not interested in anything and has no desire to do anything. She complains that she wakes up in the middle of the night and cannot sleep anymore. Even in the morning she feels worn out and without energy. She cannot concentrate on work. IN contrast to earlier, she needs much time to do anything. She hardly accomplishes her normal amount of work. She is always worried about the future and how things will continue.

Depression after the death of the husband

A few weeks ago a friend of yours lost her husband. He died in an accident. They were married ten years. Since then your friend had been practically always depressed and sad. She cannot laugh about anything anymore. She is not interested in anything and has no desire to do anything. She complains that she wakes up in the middle of the night and cannot sleep anymore. Even in the morning she feels worn out and without energy. She cannot concentrate on work. In contrast to earlier, she needs much time to do anything. She hardly accomplishes her normal amount of work. She is always thinks about her deceased husband and does not know how things can go on like this.

Depression after the partner has left

A few weeks ago, a friend of yours had her relationship ended – her partner left her. It was a complete surprise when he told her one day that he had fallen in love with another woman and wanted to live with her. Since then your friend had been practically always depressed and sad. She cannot laugh about anything anymore. She is not interested in anything and has no desire to do anything. She complains that she wakes up in the middle of the night and cannot sleep anymore. Even in the morning she feels worn out and without energy. She cannot concentrate on work. In contrast to earlier, she needs much time to do anything. She hardly accomplishes her normal amount of work. She is always thinking about the fact that her partner left her; she does not know how things can go on like this.

Depression after losing work

A few weeks ago, a friend of yours lost her job. She had worked for the same company for many years and loved her work. Since then your friend had been practically always depressed and sad. She cannot laugh about anything anymore. She is not interested in anything and has no desire to do anything. She complains that she wakes up in the middle of the night and cannot sleep anymore. Even in the morning she feels worn out and without energy. She cannot concentrate on work. In contrast to earlier, she needs much time to do anything. She hardly accomplishes her normal amount of work. She is always thinking about the job she lost and does not know how this can go on like this.

## Competing interests

All authors have no competing interests to declare.

## Authors’ contributions

MGC conceived of the study and drafted the manuscript. MCA participated in the design of the study, developed the interview and helped to draft the manuscript. HM performed the statistical analysis. AH participated in the design of the study and the development of the interview. FF helped to coordinate the survey. MFM was involved in the translation of the interview, organized the survey and supervised the interviewers. All authors read and approved the final manuscript.

## Pre-publication history

The pre-publication history for this paper can be accessed here:

http://www.biomedcentral.com/1471-244X/13/57/prepub
